# Development and Usability of a Virtual Reality-Based Filler Injection Training System

**DOI:** 10.1007/s00266-020-01872-2

**Published:** 2020-07-24

**Authors:** Seung Min Oh, Ju Young Kim, Seungho Han, Won Lee, Il Kim, Giwoong Hong, Wook Oh, Hyungjin Moon, Changmin Seo

**Affiliations:** 1 ON Clinic, Seoul, Republic of Korea; 2 Department of Family Medicine, Seoul National University Bundang Hospital, 82, Gumi-ro 173 Beon-gi, Bundang-gu, Seongnam-si, Gyeonggi-do Republic of Korea; 3 Department of Anatomy, Medical College of Choongang University, 84 Heukseok-ro Dongjak-gu, Seoul, Republic of Korea; 4 Yonsei E1 Plastic Surgery Clinic, Anyang, Republic of Korea; 5 Mania Mind CEO, Seoul, Republic of Korea; 6 SAMSKIN Plastic Surgery, Seoul, Republic of Korea; 7 Samsung Feel Clinic, Seoul, Republic of Korea; 8 BeUp Clinic, Seoul, Republic of Korea; 9 Department of Anatomy, Medical College of Choongang University, Seoul, Republic of Korea

**Keywords:** Virtual reality, Aesthetic fillers, Training, Intravascular injection, Complications

## Abstract

**Purpose:**

As filler procedures have increased in popularity, serious injection-related complications (e.g., blindness and stroke) have also increased in number. Proper and effective training is important for filler procedure safety; however, limitations exist in traditional training methods (i.e. anatomical illustrations and cadaver studies). We aimed to describe the development process and evaluate the usability of a virtual reality (VR)-based aesthetic filler injection training system.

**Materials and Methods:**

We developed the virtual reality hardware for the training system and a short guide, with a lecture regarding safe filler injection techniques. One hundred clinicians who attended a conference tested the training system. Participants completed system usability scale (SUS) and satisfaction questionnaires.

**Results:**

Nearly half of the participants were aged 35–50 years, and 38% had more than 5 years of aesthetic experience. The mean SUS score was 59.8 (standard deviation, 12.23), with no significant differences among the evaluated subgroups. Approximately 76% of participants provided SUS scores of more than 51, indicating acceptable usability. Participants aged 35–50 years were more likely to rate the system as having poor usability than were those aged < 35 years (odds ratio = 5.20, 95% confidence interval: 1.35–20.08).

**Conclusions:**

This study was the first to develop and explore the usability of a VR-based filler training system. Nearly three-fourths of participants indicated that the training system has an acceptable level of usability. However, assessments in precise target audiences and more detailed usability information are necessary to further refine the training system.

**Level of evidence IV:**

This journal requires that authors assign a level of evidence to each article. For a full description of these Evidence-Based Medicine ratings, please refer to the Table of Contents or the online Instructions to Authors www.springer.com/00266.

**Electronic supplementary material:**

The online version of this article (10.1007/s00266-020-01872-2) contains supplementary material, which is available to authorized users.

## Introduction

Aesthetic filler injection is well known as a quick or petit procedure because it can be performed quickly and easily. As a result, the proportion of filler injection cases in the cosmetic market has been rapidly increasing [1]. Accordingly, the number of cases with serious complications by the intravascular injection of the filler, such as blindness and stroke, has also increased [2]. To avoid such complications, clinical anatomic knowledge, along with technical skill, is crucial for clinicians working in plastic surgery and aesthetic medicine. Traditionally, clinicians are trained in dermal filler injection techniques during their apprenticeship via surface anatomy or cadaveric studies. However, this training results in a knowledge gap regarding the actual vessel and nerve anatomy, which is required for a safe dermal filler procedure.

Medical training systems using virtual reality (VR) equipment are actively being developed. To date, the development and the usability of VR training systems has been demonstrated in the areas of colonoscopy, psychology, arthroscopy, bronchoscopy, and surgery [3–6]. A previous study on trainees without previous anatomical knowledge showed that training with a VR anatomical atlas was associated with faster correct answers and a higher acceptance of the learning unit than that for training without the VR anatomical atlas [7]. Therefore, a filler injection training system based on VR may play a role in overcoming current training limitations in blind techniques and improving dermal filler procedure safety.

Although a VR-based filler training system has potential in improving anatomical education, the system must be evaluated by end-users in terms of its ease of use and the extent to which it satisfies the user’s needs. This evaluation should prevent future barriers in the effectiveness of the VR system. For example, usability evaluation and testing has assisted in refining the development of mobile apps for adolescent obesity management [8]. However, studies verifying the usability of a VR training system in the aesthetic medical field are lacking. Thus, it is necessary to consider the proper and effective training methods for improved filler procedure safety.

Therefore, the aim of the present study was to describe the development process and evaluate the usability of a VR-based aesthetic filler injection training system.

## Material and Methods

The present study consisted of three steps. The first step comprised the development of VR hardware for the training system, the second step comprised the creation of a short guide for clinicians, with a lecture regarding safe filler injection techniques and how to use the VR-based filler training system, and the third step comprised the actual testing of the VR-based filler training system, in which usability scores were obtained from participating clinicians. This study was approved by our Institutional Review Board (IRB number: 1041078-201902-HRBM-061-01).

### Development of the VR Hardware

**Fig. 1:**
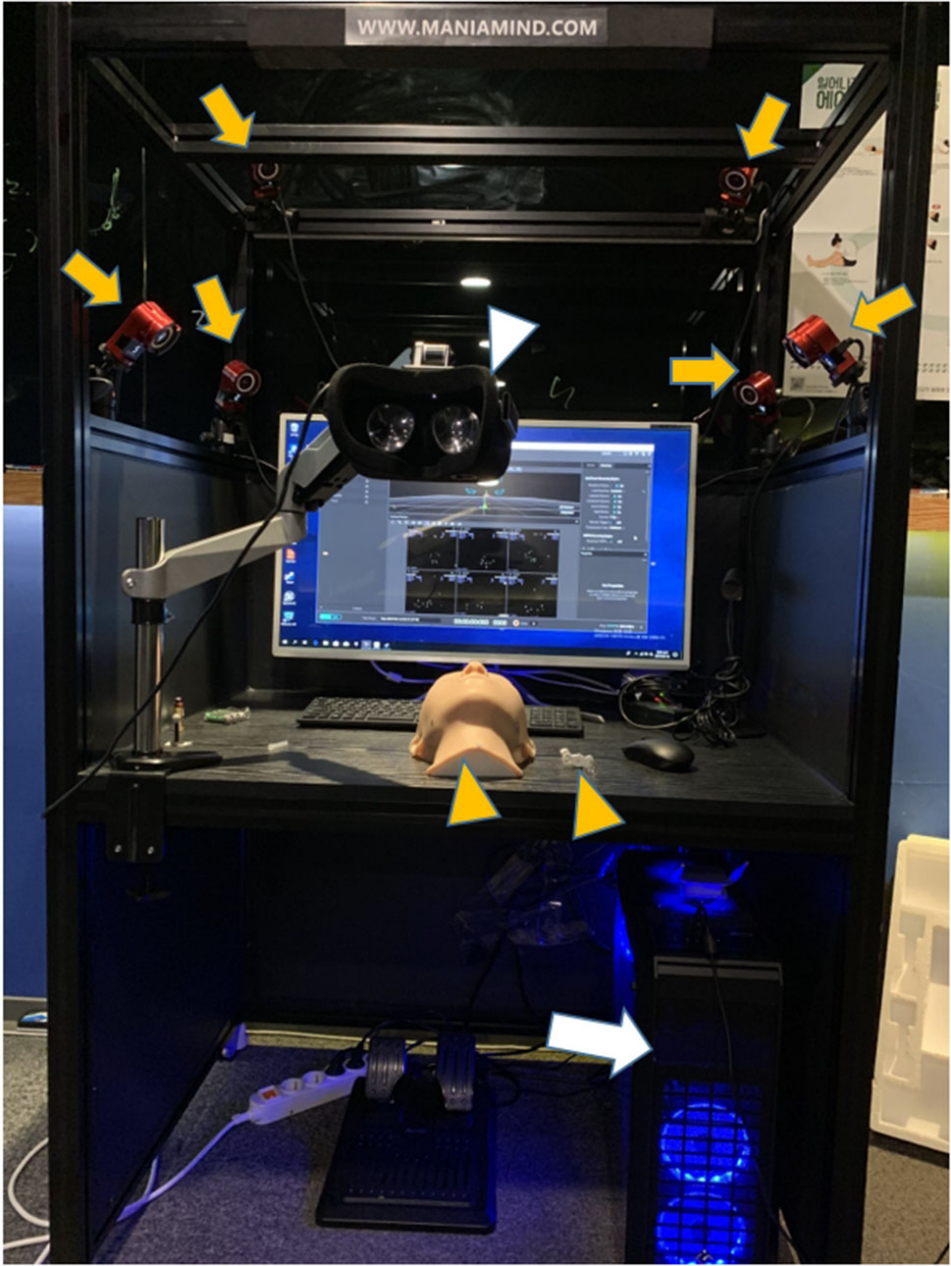
The four components of the virtual reality (VR) hardware in the VR system are shown: the main computer (white arrow), optical motion tracking system (yellow arrow), models of the human face and syringe (yellow arrow heads), and VR headset (white arrow head)

The design and development of the VR equipment was performed by Maniamind Co., Ltd. in Seoul, Republic of Korea (Fig. 1). The VR hardware consisted of four components—the main computer, an optical motion tracking system, models of the human face and syringe, and a VR headset.

**Fig. 2:**
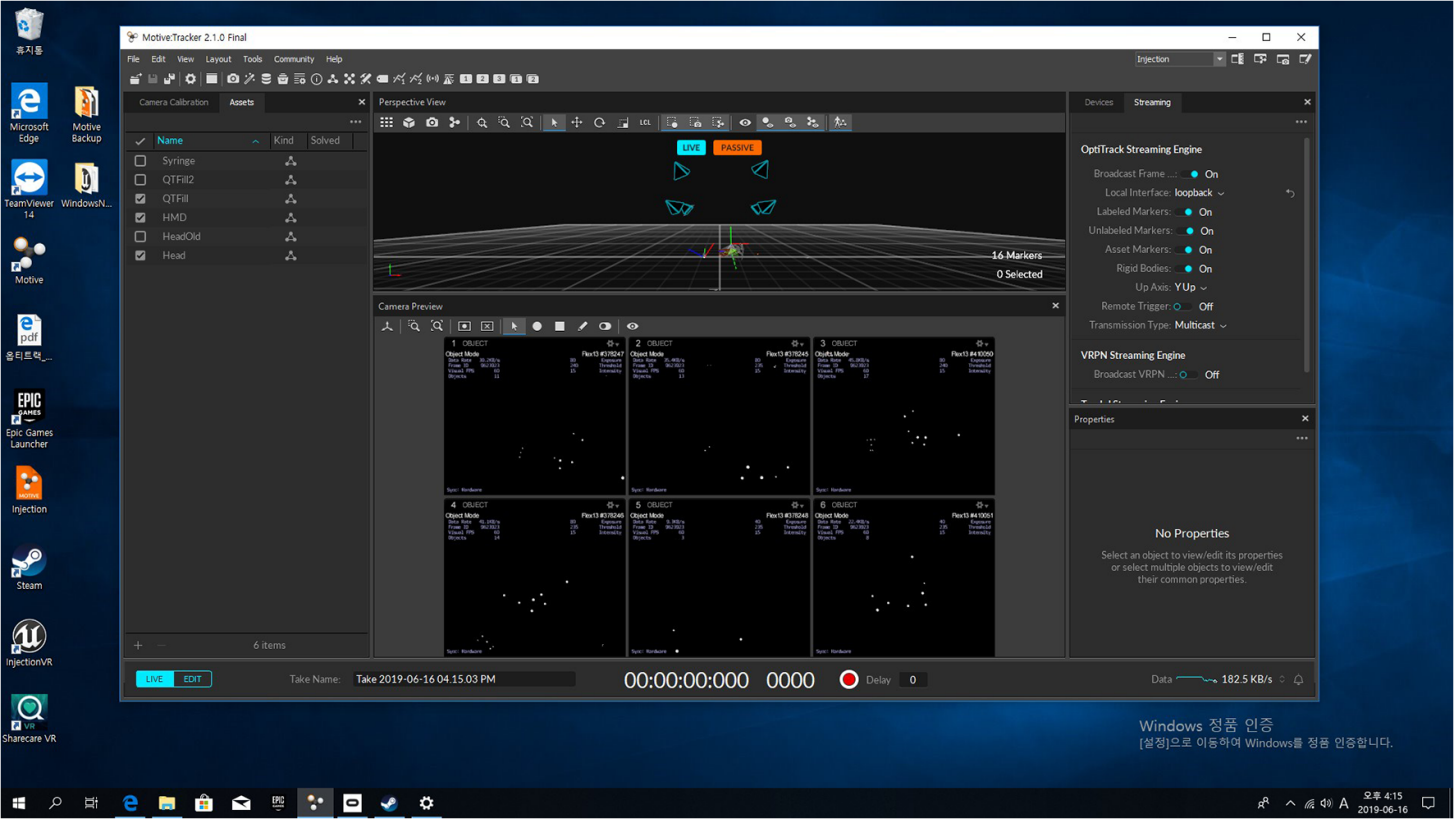
The real-time optical motion capture program (Optitrack Motive 2.1) is shown

**Fig. 3:**
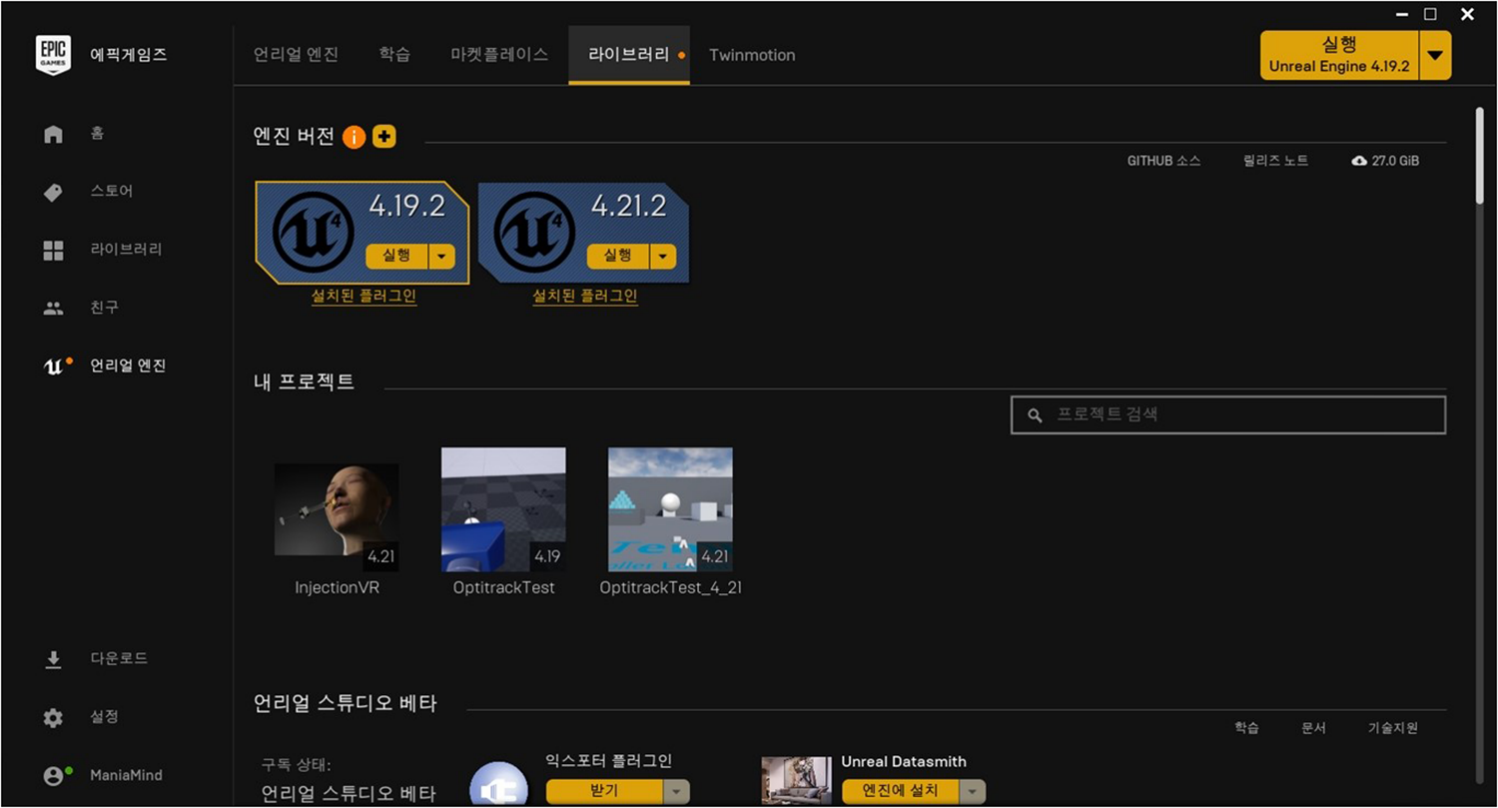
The actual training program (Unreal Engine 4.21) is shown

The main computer used Intel's i5 7500 CPU (Intel Corp, Santa Clara, California, USA) for the effective operation of the system and NVidia's GTX 1080 graphic card (Nvidia Corp, Santa Clara, California, USA) to ensure smooth real-time rendering performance. Finally, 23 GB of RAM was installed to run the program smoothly. All three-dimensional (3D) modeling data used in the training system was finally machined using 3D Max version 2017 (Autodesk Inc., San Rafael, California, USA). Motive version 2.1 (NaturalPoint Inc., Corvallis, Oregon, USA) was used to capture real-time optical motion, as shown in Fig. 2. The actual training system that effectively communicated with Motive 2.1 was made using UnrealEngine4 version 4.21 (Epic Games Company, Cary, North Carolina, USA), as shown in Fig. 3.

**Fig. 4:**
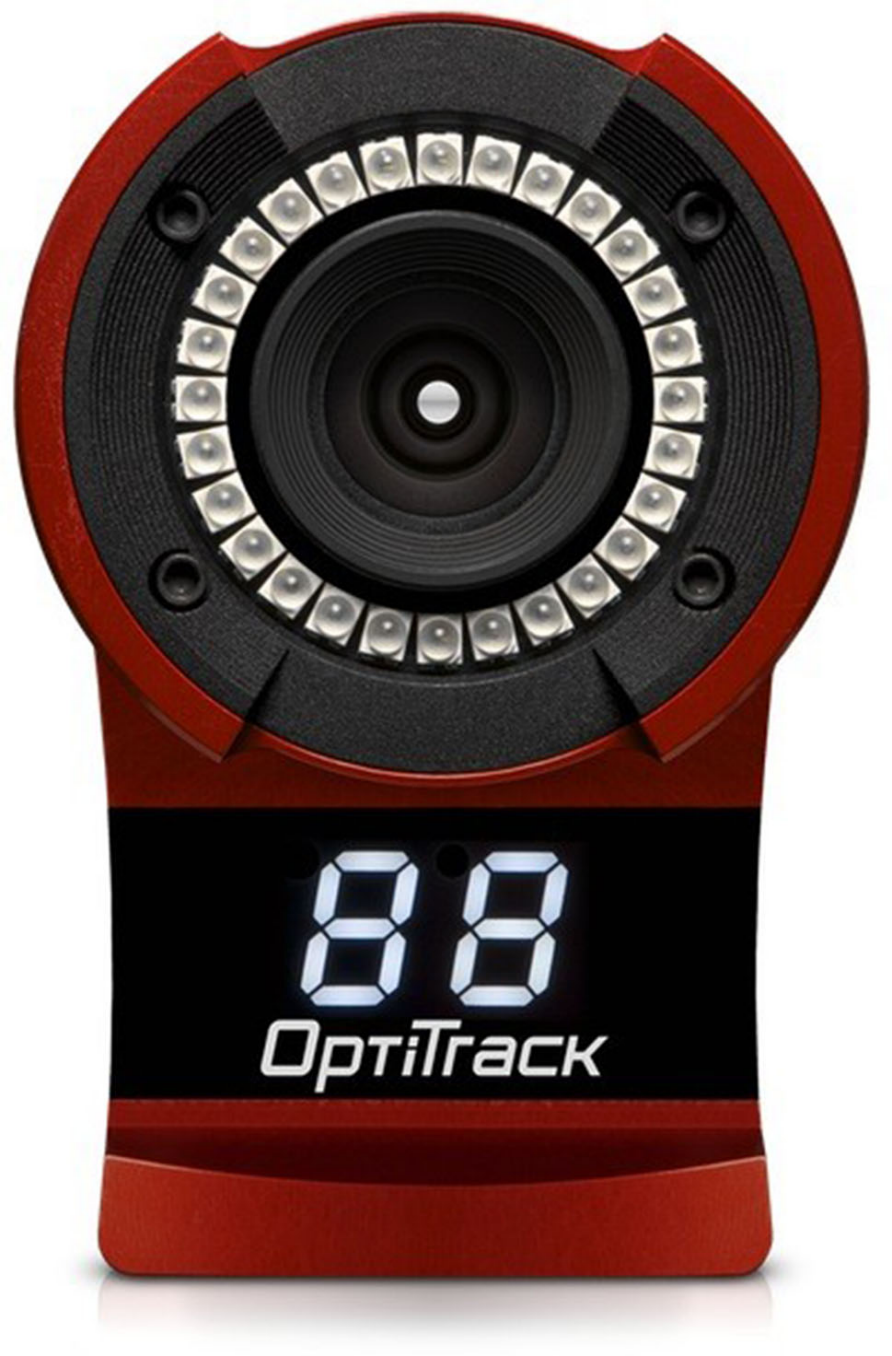
The Optitrack sensor is shown

Optical camera sets and passive markers were used for real-time motion tracking, as shown in Fig. 4. Using six Flex13 optical cameras (Optitrack, NaturalPoint Inc., Corvallis, Oregon, USA), we built an environment to track the passive markers. The fixed objects to be tracked were defined by attaching passive markers to real-space objects in patterns unique to the model of the human face and that of the syringe. As a result, the camera could recognize the face and syringe as unique independent objects in virtual space.

**Fig. 5:**
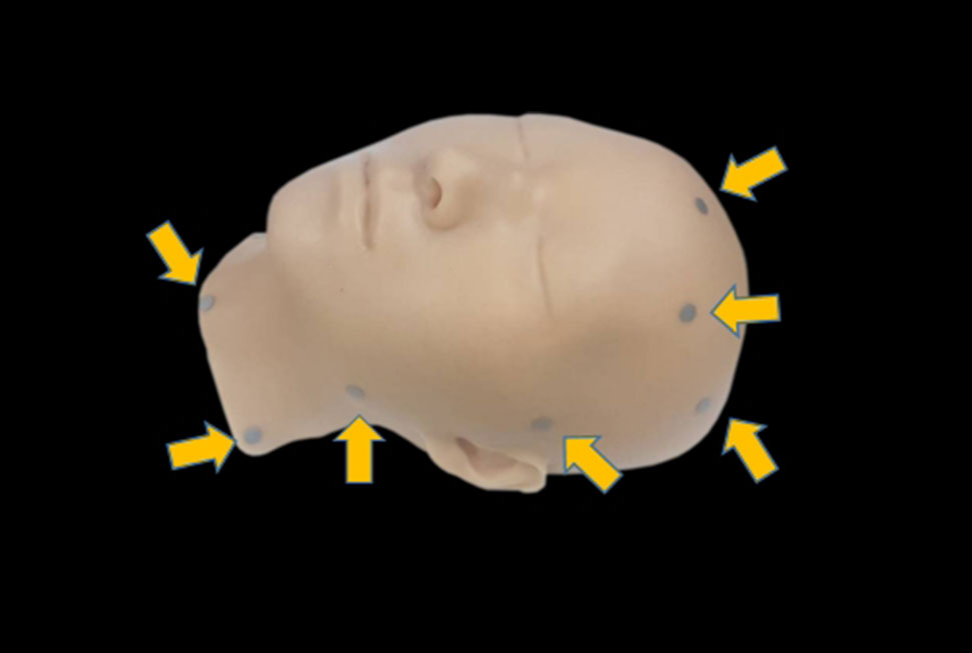
The model of the human face is shown. The model comprised the hard bony structure and soft tissue, and was made of silicon. The passive markers are also shown (yellow arrows)

The human face model, as shown in Fig. 5, was made by combining anatomical data from a cadaveric examination and radiological data from computed tomography (CT) and magnetic resonance imaging (MRI) examinations. Specially, data a from previous CT study [9] and thin-slice (1-mm interval) MRI views of a live body were combined with data from a previous research study involving a cadaveric CT scan and dissection (from the Digital Korean Human Model Database, with the kind permission of the Korea Institute of Science & Technology Information [KISTI], https://dk.kisti.re.kr/) to produce the model of the human face. The collection of these data was conducted in compliance with the Helsinki Declaration. Bone and skin data originated from the cadaveric CT scan. Soft tissue data originated from the live-body thin-slice MRI scan and live-body CT scan, as the reproducibility of the measurement of the facial soft tissue thickness is most accurate when using “perpendicular to bone” methods [9]. In such methods, the computer calculates the distance from a specific landmark in hard tissue to the point at which a vertical line intersects with the skin. In consideration of anatomical variations that can result in blindness during intravascular injection, we focused that the driving plane of vessel at a specific location is constant in spite of their variations and we decided to reflect the type of facial artery driving path most commonly found in Koreans in the human facial model [10]. Two major types of angular artery pathways (one persistent and one detouring pattern) were expressed on each side of the anterior face, based on previous anatomical research [11]. The persistent pattern, in which the angular artery originates from the branching point of the lateral nasal artery from the facial artery adjacent to the ala of the nose, was implemented on the left side. The detouring pattern, in which the angular artery traverses continuously from the detouring branch of the facial artery and ascends vertically to the nasojugal and medial canthal areas, was implanted on the right side. If we refer vascular pathway variation data according to race, gender, age, etc., we can make various human facial models based on these data. Due to the nature of the VR system, various variations can be implemented with only one human facial model using software.

The human face model can rotate up and down. Simulation is possible at the location where the operator usually performs.

**Fig. 6:**
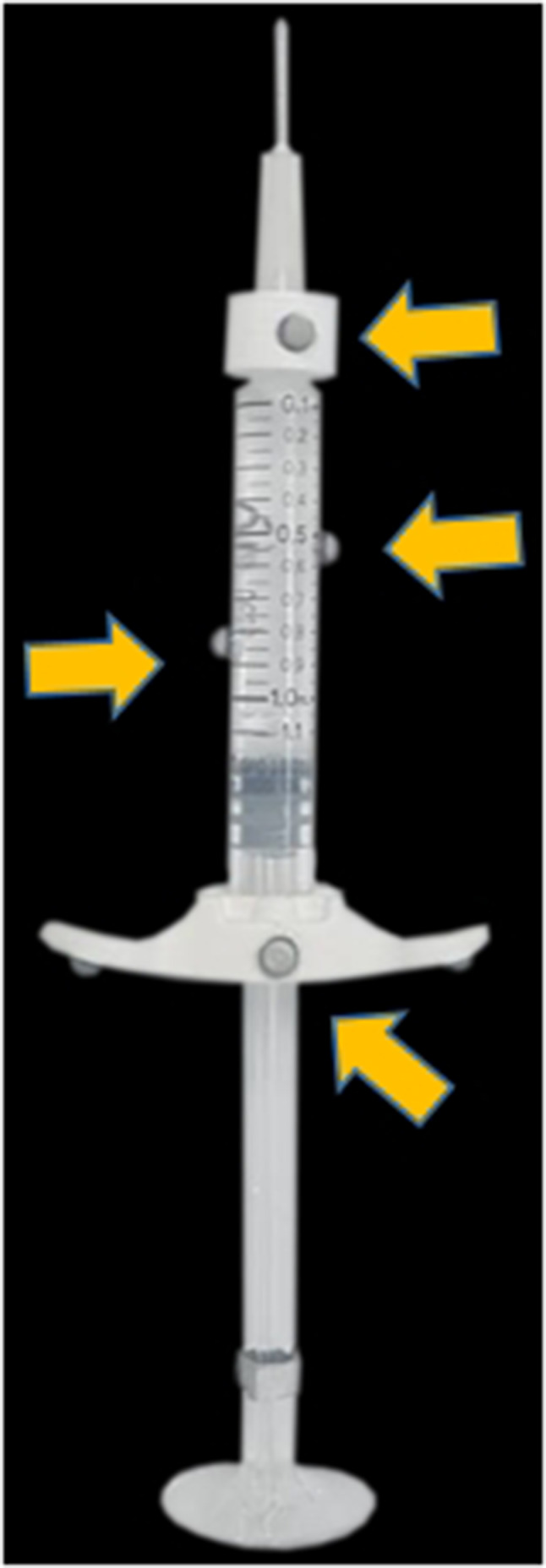
The model of the syringe is shown. An actual hyaluronic acid filler syringe was used and passive markers (yellow arrows) were attached to it. The filler product is from Sthepharm Co., Ltd. (Seoul, Republic of Korea)

The model of the syringe was created by attaching markers that the Optitrack sensor could recognize as a common syringe, as shown in Fig. 6. In the case of oblique injection or parallel injection as well as perpendicular method, it is possible to judge whether filler is injected at safe injection depth.

**Fig. 7:**
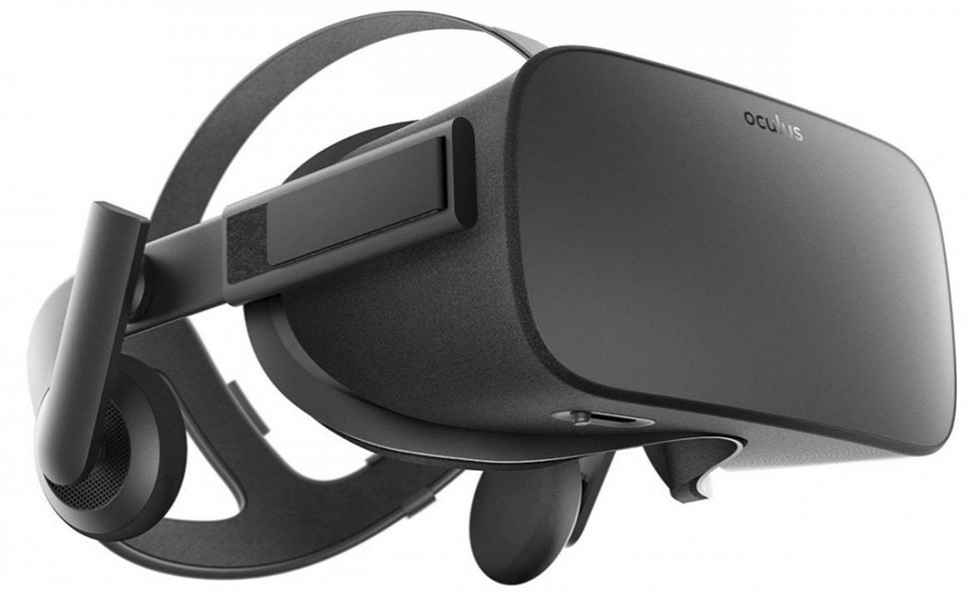
The Oculus CV1 headset is shown

Finally, we used the Oculus Rift CV1 VR headset developed by Facebook (Fig. 7).

The location of the filler injection was based on the 3D location of soft tissues, including the facial muscles and fat. Although only the skin and bones were realized in the real-space face model, all the soft tissues, such as the blood vessels, nerves, muscles, and fat, were precisely implemented in the virtual-space face model. Even if the data from the two objects (i.e., the models of the face and syringe) collided in the software, the anatomical situation could be identified. We combined the 3D fixed coordinate value of the face model with the dynamic coordinate value of the needle tip to recognize its location. Thus, it was possible to obtain 3D data of the location of the end of the needle. even if a needle of any size of 25 gauge, 27 gauge, or 30 gauge is used, whether or not the filler is injected into the blood vessel can be calculated by software. It is possible to assume that the procedure is performed using 30 gauge needle in case that the simulation is performed using 25 gaze needle and safety can be evaluated. When the tip of the needle came into contact with a nerve or vessel, an alarm system warned the practitioner that there was a possibility of injury; a red light siren rang at the top of the VR screen when a participant potentially injured blood vessels or nerves (Video, Supplemental Digital Content 1).

### A Short Guide Regarding the Safe Filler Injection Technique and How to Use the VR-Based Filler Training System for Clinicians

At the Korean Association for Laser, Dermatology, and Trichology (KALDAT) conference in Seoul on May 19, 2019, the corresponding author presented a short guide to an audience, with a lecture on safe filler injection techniques and how to use the VR-based filler training system. The author explained the visualization process of the VR-based filler injection training system for injections that use blind techniques in the real world.

After the lecture, attendants who were all medical doctors and interested in the training system were invited to enroll in the study and test the VR-based training system. After a detailed explanation of the study, interested attendees who agreed to participate in the study provided written informed consent. After study completion, a gift worth $10 USD (a skin booster) was presented to the participants.

### Testing the Usability of the VR-Based Filler Training System

**Fig. 8:**
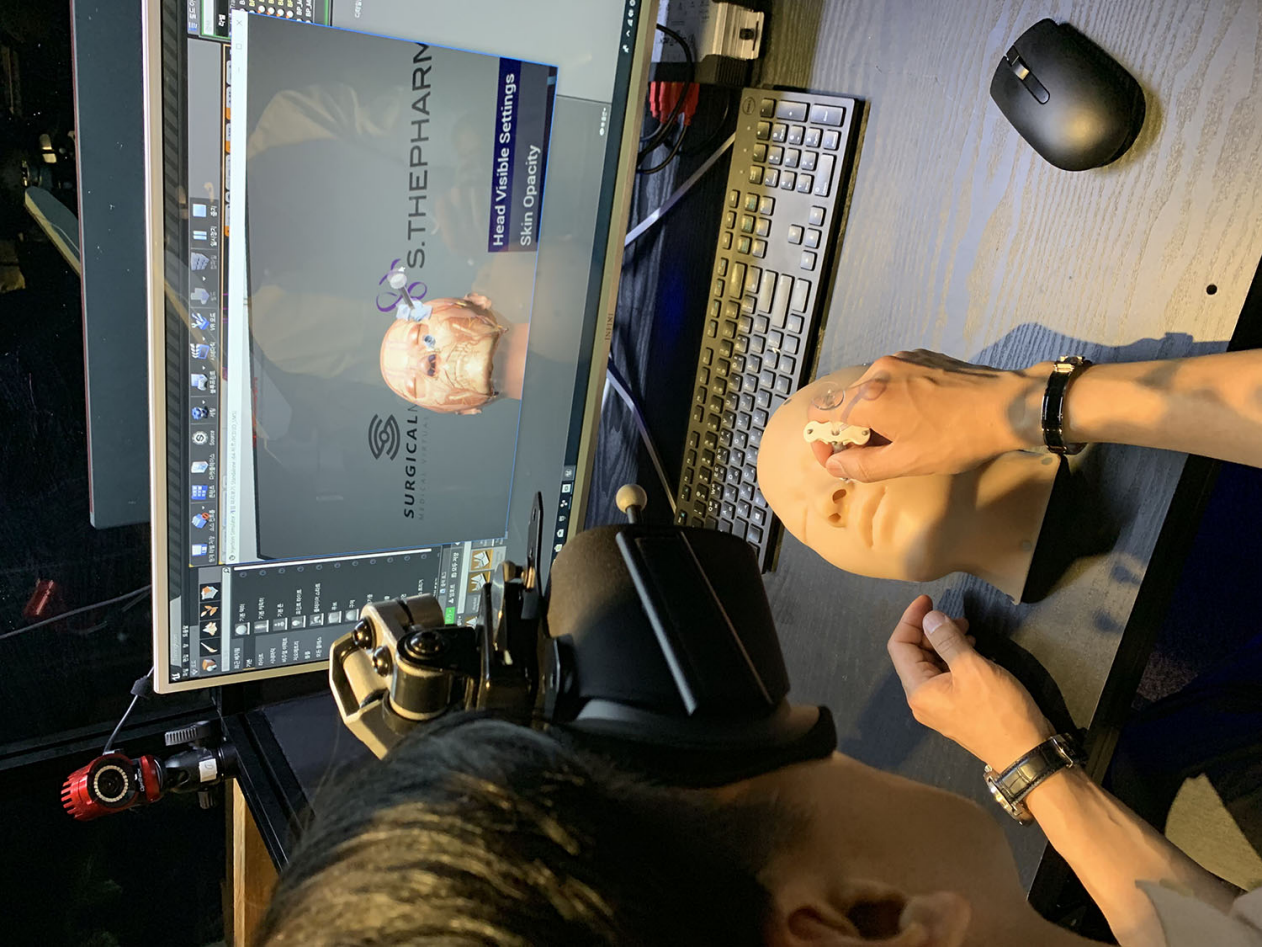
Photos of the system experience are shown

Participants (all are medical doctors) performed a filler injection procedure on the human face model while wearing the VR headset using a blind technique, as performed in their clinic (Fig. 8). Participants could see the inner structure of the face model through the VR headset. Additionally, the participants could select the inner structure that they wanted to visualize, such as vessels, nerves, superficial and deep fat, the superficial musculo-aponeurotic system, or facial muscles.

Participants simulated the filler injection procedure in the areas that they wanted to test, such as the forehead, under the eyes, anterior cheek area, or nose. Additionally, the participants checked the injection depth to determine whether the needle tip was in deep fat or superficial fat by brightening the corresponding tissue when the needle tip met areas of fat. When the filler needle tip touched vessels or nerves, the alarm system was activated and the damaged vessels or nerves were visualized. Thus, the participants evaluated the safety of their own filler injection skills.

After the task, the overall usability of the system was evaluated using the modified system usability scale (SUS) questionnaire and an additional questionnaire on subjective satisfaction. The SUS is a brief 10-item assessment of usability, which can be easily carried out, with demonstrated validity and reliability [12]. The SUS questionnaire comprises items about whether the system is easy to use, technically consistent, and suitable for continued use in the future. SUS scores of more than 50.9 are regarded as indicating acceptable usability, and scores of more than 71.4 are considered as indicating good usability [13]. The questionnaire on subjective satisfaction comprised items on whether the system aided in the identification of the main blood vessels and increased awareness of the anatomical structures requiring careful consideration during the procedure; the items were rated on 5-point Likert scales.

### Statistical Analysis

Data are summarized as means or medians for continuous variables, and as frequencies for categorical variables. Mean values from two groups were compared using the t-test and mean values from more than two groups were compared using the one-way analysis of variance. Multivariate logistic regression analyses were performed to determine the factors associated with a poor rating of the usability of the VR-based filler training system.

## Results

A total of 100 clinicians performed a filler injection procedure using the VR-based training system and completed the questionnaires. The baseline demographics of the participants are shown in Table 1. Nearly half of the participants were aged between 35 and 50 years and 38% had aesthetic experience of more than 5 years. The authors divided the medical doctors participating in the survey into aesthetics group and others group in the specialty category. The aesthetics group refers to plastic surgeons, dermatologists, and aesthetic physicians. Others are doctors who mainly treat diseases, and sometimes have cosmetic procedures such as fillers and botulinum toxins. Aesthetic experience is a question of how long the medical doctors participating in the questionnaire have been performing filler procedures.

**Table 1:** Baseline demographics of participants

Characteristics	Participants (*n* = 100)
Sex	
Male	60
Female	40
Age group	
20–35 yr	33
35–50 yr	45
> = 50 yr	17
Specialty	
Aesthetic	46
Other	53
Experience	
< 1 mo	16
1 mo-1 yr	20
1–5 yr	26
> = 5 yr	38

The mean SUS score was 59.8 (standard deviation [SD], 12.23). Mean scores according to subgroup categories, such as sex, age group, and specialties, are presented in Table 2. There were no significant differences in SUS scores among the evaluated subgroups.

**Table 2:** Mean SUS score according to subgroup

	Mean	Standard deviation	*p* value
Sex			
Male	58.71	13.16	0.1595
Female	61.44	10.64	
Age group			0.0601
20–35 yr	60.91	10.25	
35–50 yr	56.33	10.73	
> = 50 yr	63.82	17.19	
Specialty			0.637
Aesthetic	60.22	11.72	
Other	59.06	12.54	
Experience			0.7926
< 1 mo	62.03	11.41	
1 mo-1 yr	59.38	8.62	
1–5 yr	58.17	14.06	
> = 5 yr	60.2	13.09	

*SUS* system usability score

Approximately 79% of participants indicated that the VR training system was helpful in identifying the vessels and nerves requiring careful attention (mean, 4.03; SD, 0.90). Additionally, 73% of participants indicated that the VR training system helped to raise awareness, allowing a safe filler injection procedure (mean, 3.99; SD, 1.0).

Since 76% of participants rated the VR-based filler training system as having ‘acceptable’ usability (SUS score of more than 51), we explored the factors associated with a poor usability rating of our system (Table 3). Participants aged 35–50 years were significantly more likely to rate the system as having ‘poor’ usability than were participants aged less than 35 years. However, a poor usability rating was not associated with sex, years of filler injection experience, or the clinician’s specialty.

**Table 3:** Factors associated with a poor usability rating of the virtual reality-based filler training system

Factors	Odds ratio	95% confidence interval
Age between 35–50 years	5.20	1.35–20.08
Age > 50 years	2.93	0.45–19.10
Female	0.84	0.26–2.79
Aesthetic specialty	1.29	0.41–4.08
Experience > 5 years	0.30	0.04–2.19
Experience between 1–5 years	2.35	0.43–12.96
Experience between 1 month-1 year	0.47	0.07–3.27

## Discussion

To the authors’ knowledge, this study is the first to develop and explore the usability of a VR-based filler training system. Nearly three-fourths of the participants indicated that our training system has an acceptable level of usability, allows a better understanding of the anatomical location of the vessels and nerves requiring careful attention, and helps to increase awareness for a safe filler injection procedure.

The filler procedure is performed by injecting a semi-solid material into the body through a thin tube, such as a needle or cannula. The filler’s viscous and elastic properties allow it to effectively function as an implant inside the human body [14, 15]. Compared to that with traditional implantation using surgical methods, the filler procedure can be performed with less worry about scars caused by the skin incision, and additional shape correction is possible by removing the filler using an enzyme material injection [16–18]. However, the filler procedure is basically a blind technique. Thus, fillers can be incidentally injected into vessels during the procedure, which can lead to serious complications, such as blindness and stroke [2, 19, 20].

In order to avoid potentially fatal complications, clinicians are required to have a thorough, precise understanding of the anatomy of important vessels and nerves, as well as careful training based on this knowledge. General anatomical knowledge based on conventional cadaver dissections has some limitations in terms of acquiring safe procedural skills because of aging-related soft tissue changes and positional changes in live bodies. In contrast, clinicians can check the progress of the procedure while seeing blood vessels and nerves in real time using the VR-based filler training system. Moreover, the VR-based filler training system allows the conventionally blind filler technique to be visualized in a safe, controlled environment while practicing one’s skills.

When we initially developed the human face model, we wanted to implement facial soft tissue layer by layer and also implement ligament. However, the technical limitations were great. Eventually, this model was developed at the stage of distinguishing bone from soft tissue. But, safe injection depth is generally supra periosteal layer or superficial layer. Our system can clearly teach you the sense of supra periosteal layer injection and superficial layer injection. Clinicians who are new to filler procedures will find this system useful. In addition, clinicians can check whether they are injecting exactly in the intended layer or in the wrong layer.

Overall, the SUS scores indicated that the VR-based filler training system has acceptable usability. In addition, responses on the subjective satisfaction questionnaire indicated that the system is useful in increasing anatomical knowledge (mean 4.03 out of 5) and raising awareness for a safe filler injection (mean 3.99 out of 5). In a previous study on an Oculus VR system in a trauma decision-making simulator, participants and instructors had a predominantly positive response; however, performance on the course test did not significantly correlate with simulator performance in the overall candidate group [21]. Unfortunately, we did not test the participants’ anatomical knowledge, aptitude, or performance error rate during a filler injection procedure due to limits in time and budget. Instead, we utilized the short SUS questionnaire after the trial to evaluate the usability of the system, and three-fourths of the participants indicated that the system had an ‘acceptable’ level of usability. However, only three participants indicated that the system had ‘excellent’ usability, with SUS scores of more than 85.5, and 19% of participants indicated that the system had a ‘good’ level of usability, with SUS scores between 71.4 and 85.5. A previous study exploring the user-experience factors of VR systems, reported that remote controller-related factors, head tracking-related factors, and gesture-related factors are important [22]. In the present study, we did not carefully monitor or accordingly adjust the above factors to the users. Thus, further areas of improvement include the effectiveness and efficiency of the remote controller; sickness, fatigue, or immersion factors in head tracking and adaptability; simple intuitiveness; and usefulness in terms of gestures when using the VR-based filler training system.

In the present study, participants aged over 50 years or less than 35 years had more positive SUS scores compared to that for participants aged between 35 and 50 years. This might be partly due to individual differences in the level of experience and the learning curve in becoming familiar with VR technology. Participants aged between 35 and 50 years might already have better anatomical knowledge, as well as more experience in filler injections, than do participants aged less than 35 years or older than 50 years. In order to work as a medical doctor in a practice field in Korea, 11–15 years of training is required including undergraduate, internship, residentship and military service for men. In the case of the filler procedure, it is difficult to receive training during the internship or residentship course, and education is usually conducted through an external academic conference or a small seminar. So I thought that if clinicians were between 20 and 35 years of age, they would have less chance to get specialized anatomy training for filler procedure. In addition, anatomy education specialized in filler procedure, not general face anatomy, began in Korea around 2010. Therefore, it was assumed that the 35–50 years old group was more exposed to anatomy education specialized in the filler procedure through academic conferences and seminars from the beginning of the filler procedure than the group over 50 years old. Thus, the VR training system might not have met their expectations. Most VR technologies are targeted for trainees rather than experts; thus, focusing on the interests of this target audience will be a significant factor in maximizing its use as an educational tool.

The present study provides the first proof of concept, presenting an innovative VR-based filler training system with acceptable usability among clinicians in the field of aesthetic medicine. Despite several limitations, participants reported that the system was useful for learning 3D anatomical knowledge in real time, during the filler injection procedure. Moreover, we evaluated the usability of the VR-based filler training system in a relatively large number of participants compared to that in similar previous studies.

A recent review article revealed that a 3D display has benefits and shows significant potential in positively impacting anatomical education [23]. Consistent with this, the present study suggests that VR has some value in the aesthetic medical field as well. However, an assessment of the exact needs among target audiences, as well as more detailed usability information, is necessary. The system consists of a human facial model and anatomy software associated with it. It is useful for clinicians to study facial anatomy. This system is more likely to be installed in a training center, rather than clinics. Individual clinicians may use anatomy software without human face model. The price of this system is currently being set, and various usage fees will be set according to the type of use. Future well-designed randomized clinical trials are needed to determine whether the VR-based filler training system can increase the knowledge gained and reduce the error rate among clinicians.

## Conclusions

The present study was the first to develop and evaluate the usability of a VR-based filler training system. Nearly three-fourths of the participants indicated that the training system has an acceptable level of usability. Thus, we expect that the training system will be helpful for improving filler injection procedure safety; for example, training in this system could become a necessary course for licensing. However, assessments among precise target audiences and more detailed usability information are necessary to further refine the training system.

## Video, Supplemental Digital Content 1

The supplemental content shows the actual usage of the virtual reality-based filler injection training system. The alarm system is activated when the needle touches the vessels. By user selection, the desired anatomical structure can be visualized.

## Electronic supplementary material

Below is the link to the electronic supplementary material.

Supplementary file1 (MP4 6790 kb)
